# Creative Mathematical Reasoning: Does Need for Cognition Matter?

**DOI:** 10.3389/fpsyg.2021.797807

**Published:** 2022-01-07

**Authors:** Bert Jonsson, Julia Mossegård, Johan Lithner, Linnea Karlsson Wirebring

**Affiliations:** ^1^Department of Applied Education, Umeå University, Umeå, Sweden; ^2^Department of Psychology, Umeå University, Umeå, Sweden; ^3^Department of Science and Mathematics Education, Umeå University, Umeå, Sweden; ^4^Umeå Mathematics Education Research Centre, Umeå University, Umeå, Sweden

**Keywords:** algorithmic reasoning, working memory capacity, Need for Cognition (NFC), mathematical struggle, creative mathematical reasoning

## Abstract

A large portion of mathematics education centers heavily around imitative reasoning and rote learning, raising concerns about students’ lack of deeper and conceptual understanding of mathematics. To address these concerns, there has been a growing focus on students learning and teachers teaching methods that aim to enhance conceptual understanding and problem-solving skills. One suggestion is allowing students to construct their own solution methods using creative mathematical reasoning (CMR), a method that in previous studies has been contrasted against algorithmic reasoning (AR) with positive effects on test tasks. Although previous studies have evaluated the effects of CMR, they have ignored if and to what extent intrinsic cognitive motivation play a role. This study investigated the effects of intrinsic cognitive motivation to engage in cognitive strenuous mathematical tasks, operationalized through Need for Cognition (NFC), and working memory capacity (WMC). Two independent groups, consisting of upper secondary students (*N* = 137, mean age 17.13, *SD* = 0.62, 63 boys and 74 girls), practiced non-routine mathematical problem solving with CMR and AR tasks and were tested 1 week later. An initial *t*-test confirmed that the CMR group outperformed the AR group. Structural equation modeling revealed that NFC was a significant predictor of math performance for the CMR group but not for the AR group. The results also showed that WMC was a strong predictor of math performance independent of group. These results are discussed in terms of allowing for time and opportunities for struggle with constructing own solution methods using CMR, thereby enhancing students conceptual understanding.

## Introduction

A solid grasp of mathematics is a valuable life skill and a foundational goal of the Swedish national curriculum (Skolverket, 2019; The Swedish National Agency for Education). However, how to best teach and learn mathematics is a long-debated subject, both in Sweden and internationally (Loveless, 2004). A recurring concern in this debate is a lack of conceptual understanding among students for the mathematics they learn and utilize (Battista, 2001; Lithner, 2008). It is, therefore, hardly a surprise that learning and teaching methods that place a strong emphasis on conceptual understanding have been gaining more attention in the last decades (Gollub, 2002; Stylianides and Stylianides, 2007; Lithner, 2008, 2017; Shield and Dole, 2013). However, much of the mathematical education still centers around memorization and repetition, denoted as rote learning, rather than conceptual understanding (Bergqvist, 2007; Bergqvist and Lithner, 2012; Boesen et al., 2014). Indeed, in a study by Jäder et al. (2019) examining mathematic textbooks from 12 countries, it was discovered that most tasks (79%) could be solved using predefined solutions or algorithms, and an additional 13% of tasks required only minor tweaking of a previously provided template. This reliance on rote learning and imitation-based reasoning implies that when facing a task, students often problem-solve by recalling and applying algorithms they have previously memorized, based on perceived similarities to older tasks, but with little conceptual understanding of those algorithms (Lithner, 2008; Boesen et al., 2010). As a result, students reuse memorized potentially inadequate methods and thus struggle to understand why they failed or why their mathematical models did not fit (Battista, 2001).

An alternative is helping students achieve a deeper conceptual understanding by letting them create their own solution methods. Lithner (2008) presented a research framework that characterizes different types of mathematical reasoning. In this framework rote learning and imitation-based mathematical reasoning are connected to *algorithmic reasoning* (AR). Learners recall and apply previously memorized solution methods or algorithms, but with no conceptual insight or reflection on why that method should be applied. AR is contrasted with *creative mathematical reasoning* (CMR), where students create solutions when encountering new problems. CMR is defined by three criteria: (1) Novelty: the learner creates a new solution method or re-creates a forgotten one; (2) Plausibility: The learner can make arguments supporting this choice of strategy and why conclusions reached through applying the method are true or plausible; and (3) Anchoring: these arguments must be anchored in the intrinsic mathematical properties of the components used in the reasoning sequence. This process of creating a new solution implies that the learners have less support or instructions provided to them. It is argued that allowing for struggle with mathematical problems facilitates learning and develops conceptual understanding (Hiebert and Grouws, 2007; Fyfe and Rittle-Johnson, 2017). Such mathematical struggle is a key aspect of CMR (Jonsson et al., 2016).

To date, several studies have consistently found that practicing non-routine mathematical problem solving with CMR tasks is superior to practicing with AR tasks for performance on post-test assessments (Jonsson et al., 2014, 2016, 2020a; Karlsson et al., 2015; Norqvist et al., 2019b). Moreover, using transfer tasks (untrained tasks), Jonsson et al. (2020a) found empirical evidence that practicing with CMR tasks enhanced conceptual understanding of mathematics better than practicing by AR tasks. The theoretical justification is that in order to solve a task without an available solution method, it is necessary to understand the underlying mathematics, while an AR task may be solved without activating such understanding by simply following a recipe.

A critical feature of these studies has been to include measures of individual differences in cognitive abilities, such as working memory and fluid intelligence. These constructs are well-established predictors for mathematical achievement (Carroll, 1993; Floyd et al., 2003; Andersson and Lyxell, 2007; Ashcraft and Krause, 2007; Campos et al., 2013; Peng et al., 2019). The overall finding is that cognitive ability is a strong predictor of performance but is independent of practice conditions (i.e., AR or CMR; Jonsson et al., 2020a).

Another factor of importance, but which has not previously been in focus, is the role that individual differences in intrinsic cognitive motivation play in learning, here operationalized through the construct Need for Cognition (NFC; Weissgerber et al., 2018). NFC is considered a stable personality trait defined as “differences among individuals in their tendency to engage in and enjoy thinking” (Cacioppo and Petty, 1982, p. 116). NFC is not a measure of intelligence or cognitive abilities *per se* but rather a reflection of individual preference to exert more cognitive effort (Hill et al., 2016; Sandra and Otto, 2018; Weissgerber et al., 2018). NFC has been shown to predict academic achievement (Elias and Loomis, 2002) and positive associations between NFC and numerical ability have been observed (Bruine, de Bruin et al., 2015). However, the relationship between NFC and the CMR/AR distinction is unexplored. NFC is positively related to personality traits such as Openness to Experience and Conscientiousness and has repeatedly been found to have a weak to modest positive correlation to fluid intelligence, averaging around *r* = 0.20 to *r* = 0.30 (Fleischhauer et al., 2010; Furnham and Thorne, 2013; Hill et al., 2013) as well as being predictive of school success in terms of grade point average (Strobel et al., 2019). Although Hill et al. (2013) found no relationship between NFC and working memory, a follow-up study showed that working memory mediated the relationship between NFC and intelligence (Hill et al., 2016). Hill et al. (2016) argued that average working memory abilities are necessary for NFC to have a positive effect on intelligence tests. Furthermore, a study by Gonthier and Roulin (2020) found that working memory capacity (WMC) and Need for Cognition (NFC) predicted the type of strategy used on intelligence tests (Raven’s Advanced Progressive Matrices). High NFC and WMC were linked to the selection of more complex and accurate problem-solving strategies, and working memory moderated the shift toward simpler, less accurate strategies as the tasks grew more demanding. Individuals with both high NFC and WMC continued to use more complex and effective strategies throughout the tasks (Gonthier and Roulin, 2020). Albeit solving Ravens matrices is different from solving mathematical tasks it has been argued that there are many similarities between mathematical tasks typically used in schools and tasks on tests that aim to measure fluid intelligence (Blair et al., 2005).

The positive correlations between NFC, WMC and math achievements (e.g., Ashcraft and Krause, 2007; Hill et al., 2016) indicate that NFC and WMC influence math performance. Hence, as CMR tasks invoke struggle in students as a key part of the strategy’s effectiveness (Jonsson et al., 2016), how engaged and motivated a student is to struggle with CMR tasks could be an important factor in their degree of success.

Based on previous finding that cognitive ability is a strong predictor of performance but is independent of practice conditions (CMR/AR) and the assumption that practicing with CMR tasks include struggle and that high NFC is associated with more complex task solving strategies, we posed three hypotheses: (1) practicing with CMR tasks is hypothesized to be superior to practicing with AR tasks on subsequent test performance (2) WMC is hypothesized to significantly predict test performance, independent of group. (3) NFC is hypothesized to significantly predict test performance for the CMR group but not the AR group.

## Materials and Methods

In the present study, we extend a previously published experiment Jonsson et al. (2020a, experiment 1), which in turn was part of a larger data collection, including a battery of nine cognitive tests (see Jonsson et al., 2020b for a detailed description of all cognitive tasks). In Jonsson et al. (2020a, experiment 1), two independent groups of upper secondary students engaged in practicing either CMR tasks (*N* = 65) or AR tasks (*N* = 72). They solved 14 CMR and AR task sets, respectively, and were tested 1 week later on two types of practiced tasks and two types of transfer test tasks (see below for a description of both post-test practiced and transfer tasks). Moreover, measures of fluid intelligence using Raven’s Advanced Progressive Matrices (Raven et al., 2003) and a measure of complex working memory, denoted as operation span (Unsworth and Engle, 2005), were used to form a composite score of cognitive proficiency. A proficiency score that was entered together with group (AR/CMR) and math track (level of math education) as factors in a multivariate ANOVA. The multivariate ANOVA and four follow-up ANOVAs revealed significant CMR effects for all four different types of post-test tasks. The analyses also revealed a main effect of cognitive proficiency, but no multivariate group × cognitive proficiency interaction and no effect of math tracks. Hence the effect of group on the test tasks was independent of cognitive proficiency and math tracks.

From the same data set, we here extracted measures of working memory assessing WMC and NFC in conjunction with a composite score of the four test tasks as the outcome variable. Working memory is important, for example, in the selection of non-verbal problem-solving strategies. Beilock et al. (2007) found that working memory influences students’ mathematical problem-solving strategies. Working memory capacity is a key for controlling attention and inhibiting irrelevant information (Engle et al., 1999; Unsworth and Engle, 2005) and for retrieval from secondary memory (Shelton et al., 2010). Deficiencies in working memory have been connected to increased mathematical difficulties in children (Andersson and Lyxell, 2007).

### Participants

One hundred and fifty students were enrolled in the study. Six participants dropped out, and an additional seven had to be discarded due to administrative errors, so the experiment included 137 Swedish upper secondary students from the north of Sweden (63 boys and 74 girls, mean age of 17.13, *SD* = 0.62). Participants were recruited in class, from both natural science and social science programs and randomly assigned to either the AR or the CMR group. All participants were fluent in Swedish. Written informed consent was obtained from the students in accordance with the Helsinki declaration. The Regional Ethics Committee at Umeå University, Sweden, approved the study (see Jonsson et al., 2020a, experiment 1 for details). Of those 137 participants, three did not answer all items in the NFC survey and one did not respond to all tasks in the post-test. For these participants, data were replaced using regression imputation in AMOS 27.

### Materials

#### Practice Tasks

**FIGURE 1:**
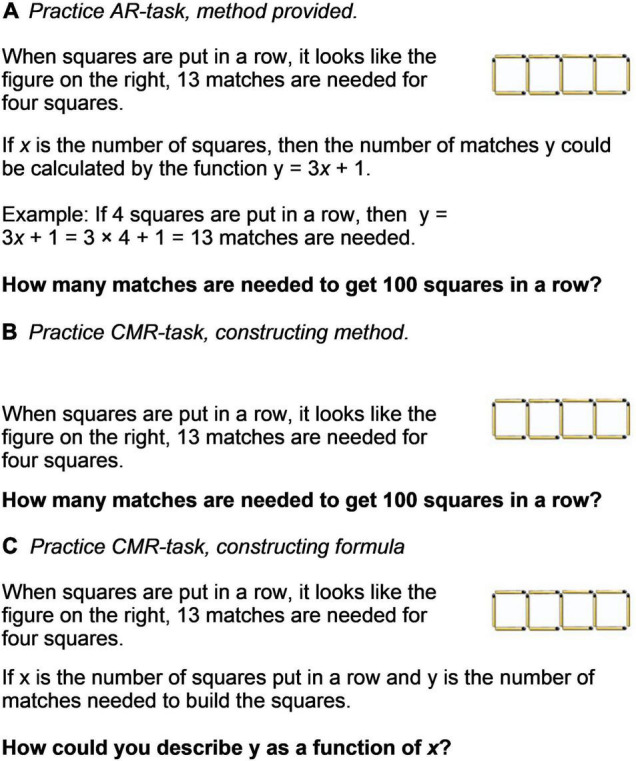
Examples of AR and CMR practice tasks and how they were presented to the students on their laptop screen. **(A)** AR practice task; **(B)** CMR tasks practice task; **(C)** CMR task asking for the formula.

The practice tasks consisted of 14 × 2 task sets of corresponding items (14 for AR and 14 for CMR, respectively). Each set had 10 sub-tasks. The practice task sets used in this study were chosen randomly from a larger pool of 28 task sets, designed to lead students toward using AR and CMR, respectively (Figures 1A,B). The AR tasks were designed to be similar to tasks found in standard mathematic textbooks. For each AR task, both a solution method (algorithm) and an example of how it should be applied were provided (Figure 1A). For the CMR tasks, no further guidance, such as an algorithm or example, was given (Figure 1B). In all CMR task sets the third subtask was to construct a formula (Figure 1C). Students were given 4 min to complete each of the 14 task sets and if a participant finished all 10 subtasks, the software randomly re-sampled new numerical tasks until time ran out. This served to make sure the AR and CMR practice conditions were equally long.

#### Post-test Tasks

**FIGURE 2:**
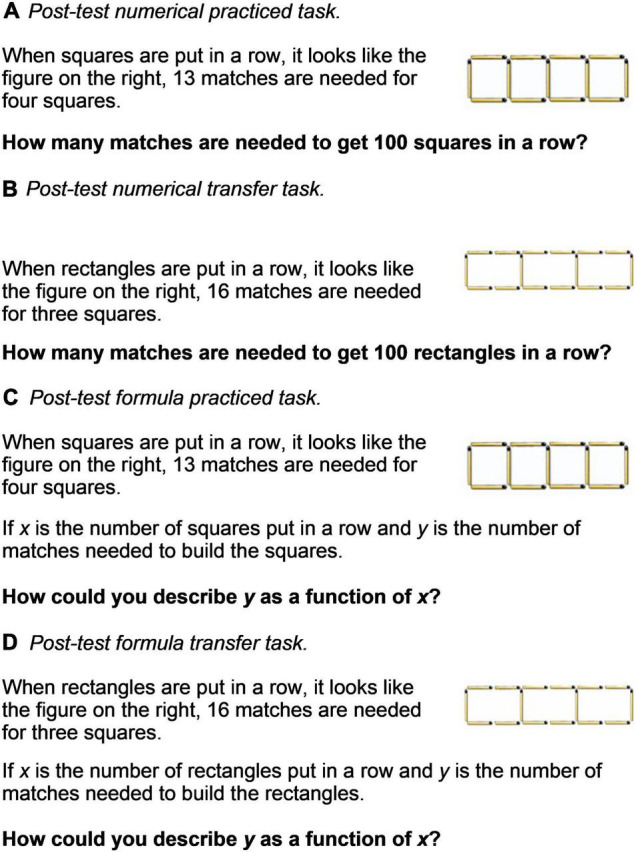
Examples of test tasks and how they were presented to the students on their laptop screen. **(A,C)** Practiced test tasks and **(B,D)** transfer test task.

There were 21 post-test tasks, 14 of which had the same layout as the CMR practice tasks (but using different numbers) and were denoted as “numerical practiced task” and “formula practiced task” (Figures 2A,C). In addition, seven tasks differed from the practice session tasks, which were denoted as “numerical transfer tasks” and “formula transfer tasks” (Figures 2B,D). The transfer post-test tasks shared underlying solution ideas with the practice tasks, but could not be solved using the same formulas. The distinction between transfer test tasks and practiced test tasks is further described in Jonsson et al. (2020a; experiment 1). The time limit for the post-test tasks was 4 min. More extensive descriptions of both practiced tasks and test tasks can be found in Jonsson et al. (2014), Norqvist et al. (2019a), and Jonsson et al. (2020a) as well as in Supplementary Material provided with the Jonsson et al.’s (2020a) study.

#### Working Memory Measures

The working memory measures included were operation span (Unsworth and Engle, 2005), block span, and digit span assessing the central executive, spatial short term memory and phonological short term memory, respectively (Baddeley, 2003). In the operation span task, participants are instructed to do mathematical calculations. After each calculation, they are asked to maintain a letter (displayed for 800 ms) in their memory. They are then presented with a new mathematical task and asked to maintain both the previous and the new letter in their memory. After a full set is completed (each set contains three to seven letters), the participants are asked to identify the letters in the order they were presented. There were three sets of each size and the participants score was the sum of all correctly recalled sets (Unsworth and Engle, 2005). Operation span was administered via computer and self-paced. In the block span task participants were instructed to remember squared blocks presented on a computer screen in 4 × 4 matrices separated by an interstimulus interval of 1 s. The squares were presented as sequences of squares increasing in difficulty—from two squares, three squares, four squares…. up to a limit of 16 squares. After a delay of 2 s participants were prompted to tap on the squares in the same order as they were presented. The total number of perfectly recalled sequences was used as the dependent variable. In the digit span task, numbers between 1 and 9 were presented on the computer screen in random order with an interstimulus interval of 1 s. After a delay of 2 s, participants were prompted to recall the numbers in the same order as they were presented. The test started with a two-digit sequence and increased by one digit as long as the participants managed to repeat the correct sequence. The highest sequence length was used as dependent measure., See Jonsson et al. (2020b) for extensive descriptions of the tasks and their psychometric properties.

#### Need-for-Cognition

Need for Cognition was measured by the *Mental Effort Tolerance Questionnaire* (METQ; Dornic et al., 1991), a Swedish adaptation of the original NFC Scale by Cacioppo and Petty (1982). The METQ consists of 30 items that are rated on a 5-point Likert-like scale (from 1 = strongly disagree to 5 = strongly agree). 12 items indicate positive and 18 items negative attitudes toward engaging in cognitive activity. The negative attitude items are scored reversely. An example of a positive attitude item from the METQ scale is “It is important to ponder upon why things work as they do” (Dornic et al., 1991, p. 316). Stenlund and Jonsson (2017) evaluated the psychometric properties of the 30-item METQ scale and found good internal consistency (α = 0.88) and test-retest reliability (*r* = 0.88). The high internal consistency and high test-re-test reliability indicate that the full 30 item NFC scale is a valid and reliable measure.

The working memory tasks and METQ, were selected due to their known associations with math performance and mathematical problem-solving strategies tasks (e.g., Beilock et al., 2007; Gonthier and Roulin, 2020) as well as their good psychometric properties. See Jonsson et al. (2020b) for extensive descriptions of the tasks and their psychometric properties.

### Procedure

In a between-group design, the participants were randomly assigned to either the AR or CMR groups (*N* = 72 and 65, respectively). The working memory measures and the NFC survey were completed 1 week before the practice session, and there was 1 week between the practice and post-test sessions.

During the training session students worked individually, receiving mathematics tasks and submitting answers through a computer. We recognize that both cooperative and individual learning can be valuable (e.g., Cross, 2009; OECD, 2017; Parveen et al., 2017) in ordinary classrooms. The individual approach in this study was motivated by the ambition to link individual measures of working memory and NFC to mathematical practice and posttest performance. After a student submitted an answer, the correct answer was displayed. No such feedback was given after the formula construction tasks (the third CMR task). This was to prevent the CMR task from turning into an AR task, as the students could then memorize the formula and apply it to later subtasks instead of constructing their own solution.

For the post-test session, the practiced and transfer tasks could be further split into numerical and formula tasks. In the formula tasks for both practiced and transfer tasks, the students were asked to write down the formula (Figures 2C,D). The practiced tasks were presented before the transfer tasks. The first task for both practiced and transfer tasks was a formula task and the second a numerical task.

Both the practice and the post-test sessions were conducted in the students’ classroom. No teacher or peer support was available, but the students were offered the assistance of a simple virtual calculator displayed on the screen of their laptops. The software used automatically corrected and saved the students’ answers during both the practice and post-test sessions. For additional examples and descriptions of the tasks used in this study, see Norqvist et al. (2019a).

### Statistical Analyses

In Jonsson et al. (2020a, experiment 1) the statistical analyses showed that training with CMR tasks was superior to training with AR tasks on all four types of test tasks: retrieving the formula from memory for both practiced- and transfer tasks and solving numerical practiced- and transfer tasks. In order to reduce the number of models, we collapsed the four test tasks (two practiced and two transfer tasks) used in Jonsson et al. (2020a, experiment 1) to a composite overall measure of performance, denoted as composite test performance (C-TP).

The working memory measures were used as indicators of a latent WMC factor, while the items in the NFC scale, with a Chronbachs alpha of 0.89, were collapsed to form a composite score of NFC.

First, the descriptive information of the study sample was summarized followed by zero-order correlations between all the variables included in the analyses (see Tables 1, 2). Second, to confirm the AR-CMR group differences found in Jonsson et al. (2020a, experiment 1), an initial *t*-test of the composite test scores was conducted. Third, three structural equation models (SEM) investigated the effects of WMC and NFC on C-TP (the dependent variable). The first model included all participants, the second and third analyzed CMR and AR groups separately. Due to the known correlation between WMC and NFC (e.g., Stenlund and Jonsson, 2017; Gonthier and Roulin, 2020), the models covary the latent factor WMC with NFC. Three fit indices were used to evaluate the models, including the comparative fit index (CFI), the root mean square error of approximation (RMSEA), and χ^2^ divided by degrees of freedom. To attain an acceptable fit for CFI, the value must be equal to or greater than 0.95 (Browne and Cudeck, 1989). RMSEA values need to be equal to or less than 0.06 to attain a good model fit and 0.08 for a reasonable fit (Browne and Cudeck, 1989; Hu and Bentler, 1999). Note that the sample sizes used in the group specific analyses could be regarded as low (Kline, 2013). However, Tanaka (1987) argued that a sample size of 50 could be enough when the model is simple. The models in this study contain only one exogenous latent factor, one exogenous manifest variable and one endogenous variable. The data were analyzed using SPSS (IBM Corporation, Armonk, NY, United States) and AMOS 27 (Arbuckle, 2016) with bias-corrected percentile method as bootstrapping procedure.

**TABLE 1:** Descriptive statistics for the continuous variables.

	C-TP	Operation span	Block span	Digit span	NFC
CMR	0.297 (0.216)	32.231 (16.668)	13.754 (2.616)	3.108 (1.047)	102.776 (16.340)
AR	0.179 (0.182)	30.875 (16.152)	13.466 (2.959)	3,278 (1,224)	99.139 (14.674)

*Mean values with standard deviation in the parentheses. CMR, Creative Mathematical Reasoning group; AR, Algorithmic Reasoning group: C-TP, Composite Test Performance; NFC, Need for Cognition.*

**TABLE 2:** Pearson’s correlations.

Variables	1	2	3
1. O span	—		
2. Digit span	0329***	—	
3. Block span	0.388***	0.185*	—
4. NFC	0.301***	0.190*	0.08P^ ns^

***p < 0.0001; *p < 0.05.

### Ethical Considerations

The data used in this study were obtained as part of a research project that has been approved by the Regional Ethical Review Board in Umeå. The process of collecting the data followed current principles and guidelines as specified by the Swedish Research Council. Written informed consent was obtained from each participant.

## Results

Descriptive statistics and correlations between the continuous variables can be seen in Tables 1, 2, respectively. All continuous variables were approximately normally distributed, with values below 0.81 for both skewness and kurtosis. No values outside a third interquartile range were detected. *T*-tests confirmed that the groups were equal with respect to NFC, operation span, digit span and block span, all *p*’s > 0.17), meaning that the two groups can be considered to be equal when it comes to working memory and NFC. The correlations were significant, except for the correlation between block span and NFC (see Table 2). The initial *t*-test confirmed as expected that participants in the CMR group outperformed their counterparts in the AR group *t*(135) = 3.44, *p* < 0.001.

**FIGURE 3:**
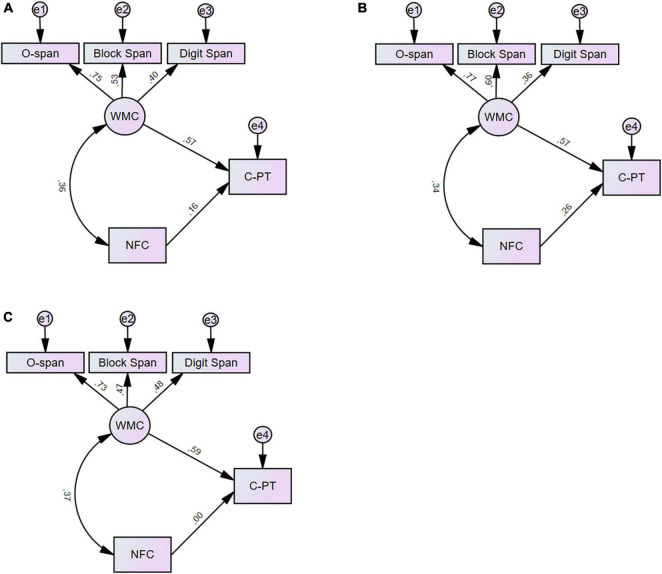
The figure shows the standardized regression weights for both groups **(A)**, CMR **(B)** and AR **(C)**, separately. C-PT, composite test performance; WMC, Working memory capacity; NFC, Need for Cognition.

Figures 3A–C shows the SEM models with regression weights for the overall model (a), the CMR group (b) and the AR group (c), separately. The results of standardized and unstandardized beta weights, standard error and *p*-values from the SEM analyses accompanied by bootstrapping (95% CI) and *p*-values can be seen in Table 3. The overall model indicated reasonable fit with CFI = 0.975, RMSEA = 0.066, χ^2^/df = 1.60, *p* = 0.172, explaining 42% of the variance for C-TP. The model fit for CMR was excellent; CFI = 1.00, RMSEA = 0.000, χ^2^/df = 0.81, *p* = 0.516, explaining 50% of the variance for C-TP. Model fit was a bit lower for AR; CFI = 0.925, RMSEA = 0.105, χ^2^/df = 1.78, *p* = 0.129, explaining 35% of the variance for C-TP. The direct effect of WMC on C-TP was almost identical across groups. The most apparent difference was that the NFC > C-TP path was significant for the CMR model (β = 0.26) but not for the AR model (β = 0.00) (see Table 3 for details). However, constraining the NFC > C-TP path and performing a Boostrapping, bias-corrected percentile significant test did not reach a significant between group difference, *p* = 0.15.

**TABLE 3:** Path analyses with test task performance as dependent variable.

					Bootstrapping (BC 95% CI)		
*Overall*	β	*B*	S.E	*p*	Lower	Higher	*p*
WMC → C-TP	0.571	0.259	0.076	<0.001	0.132	0.576	0.001
NFC → C-TP	0.163	0.002	0.001	0.059	0.000	0.005	0.108
WMC → Digit span	0.400*						
WMC → O-span	0.749	26.773	7.364	<0.001	17.035	53.329	0.001
WMC → Block span	0.533	3.255	0.951	<0.001	1.742	6.849	0.001
**CMR group**							
WMC → C-TP	0.572	0.325	0.141	0.021	0.148	1.049	0.002
NFC → C-TP	0.263	0.003	0.002	0.021	0.000	0.008	0.037
WMC → Digit span	0.364*						
WMC → O-span	0.774	33.801	13.963	0.015	15.498	160.732	0.001
WMC → Block span	0.601	4.120	1.756	0.019	1.729	16.225	0.001
**AR group**							
WMC → C-TP	0.594	0.185	0.068	0.007	0.072	1.186	0.005
NFC → C-TP	0.003	0.000	0.002	0.980	–0.004	0.003	0.970
WMC → Digit span	0.478*						
WMC → O-span	0.735	20.256	6.781	0.003	10.382	52.447	0.001
WMC → Block span	0.601	2.371	0.914	0.009	0.679	10.786	0.013

BC, bias corrected; 2,000 bootstrap samples; β, Standardized regression weight; B, Unstandardized regression weight; P, Significance of Estimates; *Constrained parameter.

## Discussion

How to help students better develop a conceptual understanding of mathematics is under scrutiny and is regarded as an important question (e.g., Loveless, 2004; Lithner, 2008, 2017). One suggested solution is to help students build conceptual understanding by constructing their own solution methods, denoted using *CMR* (Lithner, 2008, 2017). CMR is often contrasted against the more common method based on imitative reasoning, *AR*. Several previous publications have shown that practicing with CMR tasks when students construct the solution is superior to AR (Jonsson et al., 2014, 2016, 2020a; Norqvist et al., 2019b). However, to what extent intrinsic cognitive motivation influences performance has not been investigated. Here we extended a previous publication (Jonsson et al., 2020a, experiment 1) by assessing the influence of NFC and WMC on math performance independent of group and separately for CMR and AR groups. To reduce the number of parameters, we collapsed the four dependent measures used in Jonsson et al. (2020a, experiment 1) to a composite score (C-TP), assessing participants overall performance. The initial analyses of the psychometric properties showed that all continuous variables were normally distributed and that the groups were equal regarding the cognitive ability measures and NFC. In line with previous studies, it was hypothesized that practicing with CMR tasks should be superior to practicing with AR tasks on subsequent test performance. It was hypothesized that NFC would be predictive of performance for the CMR group but not for the AR group. It was also hypothesized that WMC would predict performance for both groups.

The initial *t*-test of group difference based on the composite score of the four dependent variables used in Jonsson et al. (2020a, experiment 1) was significant. Hence participants in the CMR group outperformed their counterparts in the AR group, as indicated in Table 1, confirming hypothesis 1. This result also replicated other previous findings (Jonsson et al., 2014, 2016; Norqvist et al., 2019b), adding to a growing pile of evidence showing the positive effects of encouraging students to train creative mathematical reasoning.

The second hypothesis was confirmed, showing that the measure of NFC did predict mathematical performance following CMR—but not AR training. This finding is in line with the argument that NFC support selection of more complex and accurate problem-solving strategies (Rudolph et al., 2018; Gonthier and Roulin, 2020). To note is that the group comparison for the NFC > C-TP path did not reach significance. However, it seems likely that this is a question of power. In addition, in all three SEM analyses, we covary NFC and WMC, thereby controlling for the combined effects of NFC and WMC.

The third hypothesis, that WMC would predict mathematical performance on the post-test independent of group was also confirmed. The main effect of WMC is in line with established research on the effects of cognitive abilities on mathematical performance (e.g., Campos et al., 2013; Peng et al., 2019). The fact that the effect of WMC was obtained independent of group indicates that using CMR is not only for the cognitively stronger students. However, the positive correlation between WMC, and NFC, and the effect of NFC on CMR tasks implies that the motivation to engage in cognitively strenuous tasks is higher among those with higher WMC. From a didactical perspective, it is therefore critical to allow, provide time, and encourage all students to struggle with mathematical problems to create their own task solutions. Thereby, CMR training could be accessible and effective even for students who lack the motivation to engage in cognitively strenuous mathematical tasks.

### Limitations and Future Research

The psychometric properties, tight SEM models, and hypothesis-driven analyses are strengths. With that said, the significant effects of NFC must be interpreted with caution, partly due to the relatively low sample size and that this is the first study that focused on NFC and creative mathematical reasoning. Another important note is that the sample was restricted to upper secondary students. Since NFC is known no develop over time, and the correlation with WMC is relatively high, the external validity in terms of generalizability to younger students is difficult to assess.

We hope that this first study on the influence of intrinsic cognitive motivation regarding creative and algorithmic mathematical reasoning will encourage researchers to conduct more studies. Considering the developmental paths of both NFC and cognitive ability, a longitudinal within-subject approach would be desirable.

Although more research is needed, we emphasize the need to provide time and opportunities for struggle with creative mathematical tasks, thereby enhancing students conceptual understanding. With that said, we have in previous studies discussed the potential of combining the CMR approach with other validated methods that are designed to facilitate mathematical understanding, such as worked example, self-explanation and retrieval practice. Regarding retrieval practice and CMR, we have in a recent publication (Stillesjö et al., 2021) demonstrated common neurocognitive long-term memory effects by using functional magnetic brain imaging (fMRI). The brain imaging data indicate that active learning conditions, such as CMR and retrieval practice engage a shared brain network with higher functional brain activity for these learning methods when compared to more passive such as re-study and AR, despite dissimilar study material (math problems for CMR and Swahili vocabulary for retrieval practice). These findings are argued to be related to the formation and reactivation of semantic representations and raise the question and potential of combining retrieval practice with CMR. It is also interesting to discuss the potential to integrate CMR with cooperative learning. Indeed, an initial study focusing on collaborative learning using CMR tasks has, as pointed out above, been conducted (Granberg and Olsson, 2015). Designing situations which invite to cooperative struggle with CMR tasks seems feasible and a productive way to move forward. However, the effects of combining CMR with retrieval practice or cooperative learning is at the end of the day an empirical question.

## Conclusion

In summary, this study demonstrates that training with CMR tasks yields better mathematical performance than AR tasks and that cognitive abilities strongly affect mathematical performance independent of group. These results add to a stable pattern of CMR, showing good effects on mathematical performance and strengthening its viability as an educational strategy. Although WMC was a significant and robust predictor, the effects were equally strong in both groups. The influence of NFC on performance for those that had practiced with CMR tasks seems logical in relation to the structure of CMR tasks and the NFC construct.

## Data Availability Statement

The raw data supporting the conclusions of this article will be made available by the authors, without undue reservation.

## Ethics Statement

The studies involving human participants were reviewed and approved by the Regional Ethics Committee at Umeå University. Written informed consent from the participants’ legal guardian/next of kin was not required to participate in this study in accordance with the national legislation and the institutional requirements.

## Author Contributions

BJ, LK, JL, and JM came up with the idea for the study and jointly contributed to the study’s conceptualization, and revised the manuscript for important intellectual content. BJ performed the statistical analysis and wrote the first draft of the manuscript, whereby all authors contributed to the manuscript and read and approved the submitted version.

## Conflict of Interest

The authors declare that the research was conducted in the absence of any commercial or financial relationships that could be construed as a potential conflict of interest.

## Publisher’s Note

All claims expressed in this article are solely those of the authors and do not necessarily represent those of their affiliated organizations, or those of the publisher, the editors and the reviewers. Any product that may be evaluated in this article, or claim that may be made by its manufacturer, is not guaranteed or endorsed by the publisher.
